# Monitoring and Control Interface Based on Virtual Sensors

**DOI:** 10.3390/s141120645

**Published:** 2014-10-31

**Authors:** Ricardo F. Escobar, Manuel Adam-Medina, Carlos D. García-Beltrán, Víctor H. Olivares-Peregrino, David Juárez-Romero, Gerardo V. Guerrero-Ramírez

**Affiliations:** 1 Centro Nacional de Investigación y Desarrollo Tecnológico, Tecnológico Nacional de México, Int. Internado Palmira S/N, Palmira, C.P. 62490 Cuernavaca, Morelos, Mexico; E-Mails: esjiri@cenidet.edu.mx (R.F.E.); adam@cenidet.edu.mx (M.A.-M.); cgarcia@cenidet.edu.mx (C.D.G.-B.); gerardog@cenidet.edu.mx (G.V.G.-R.); 2 Centro de Investigación en Ingeniería y Ciencias Aplicadas, Universidad Autónoma del Estado de Morelos, Av. Universidad 1001, Col. Chamilpa, C.P. 62209 Cuernavaca, Morelos, Mexico; E-Mail: djuarezr7@gmail.com

**Keywords:** monitoring and control interface, virtual sensors, interactive educational workstation, heat exchanger

## Abstract

In this article, a toolbox based on a monitoring and control interface (MCI) is presented and applied in a heat exchanger. The MCI was programed in order to realize sensor fault detection and isolation and fault tolerance using virtual sensors. The virtual sensors were designed from model-based high-gain observers. To develop the control task, different kinds of control laws were included in the monitoring and control interface. These control laws are PID, MPC and a non-linear model-based control law. The MCI helps to maintain the heat exchanger under operation, even if a temperature outlet sensor fault occurs; in the case of outlet temperature sensor failure, the MCI will display an alarm. The monitoring and control interface is used as a practical tool to support electronic engineering students with heat transfer and control concepts to be applied in a double-pipe heat exchanger pilot plant. The method aims to teach the students through the observation and manipulation of the main variables of the process and by the interaction with the monitoring and control interface (MCI) developed in LabVIEW^©^. The MCI provides the electronic engineering students with the knowledge of heat exchanger behavior, since the interface is provided with a thermodynamic model that approximates the temperatures and the physical properties of the fluid (density and heat capacity). An advantage of the interface is the easy manipulation of the actuator for an automatic or manual operation. Another advantage of the monitoring and control interface is that all algorithms can be manipulated and modified by the users.

## 1. Introduction

It has been observed that due to the academic training received by electronic engineering students, it is difficult for them to understand heat transfer concepts and how to deal with cases in which a failure occurs. Therefore, at the National Center for Research and Technological Development (CENIDET (Centro Nacional de Investigación y Desarrollo Tecnológico)), a monitoring and control interface (MCI) for a double-pipe heat exchanger was developed. The MCI is orientated to provide support for the electronic engineering students, and so, the MCI has two main purposes. On the one hand, this system provides the students with the knowledge of heat exchanger behavior. The MCI shows on-line the physical property behavior of the fluid in the double pipe heat exchanger *ρ* (density) and *C_p_* (heat capacity). Additionally, the MCI shows on-line the value of the convective heat transfer coefficients (*h_o_, h_i_*) and the global heat transfer coefficient (*U*), which are calculated by a thermodynamic model. On the other hand, the information obtained through the thermodynamic model is used by a nonlinear model that describes the temperature dynamics in the double pipe heat exchanger. A fault-tolerant system-based model was implemented as a practical example. The MCI allows manipulating the system actuators in order to perform control tasks. The interface can be operated in open loop or in closed loop. The main objective of the monitoring and control interface is to provide the students with educational workstation with practice in the control area. This work represents the effort of the lecturers who developed the educational workstation based on their own research, which is implemented in the heat exchanger pilot plant.

The main objective of the industry is to keep processes operating adequately; nevertheless, nowadays, the industry demands constant monitoring of the systems in order to improve the reliability and safety of the involved processes. Thus, monitoring systems must be efficient and capable of supervising the main variables of the process and to implement control tasks in order to enhance user capabilities. Industrial processes, such as mining, chemical, water treatment, among others, use complex systems that operate in different regimes, which make them difficult to control. Therefore, this work was developed in order to prepare electronic engineering students (EES) for these challenges and to give them multidisciplinary training.

Heat exchangers are widely used in power production, process, chemical and food industries, electronic, environmental engineering, waste heat recovery, manufacturing industries, space heating, refrigeration, air conditioning, chemical plants, petrochemical plants, petroleum refineries and natural gas processing. Therefore, it is extremely important for EES to learn the techniques of process control focused on heat exchangers.

Mathematical algorithms are becoming widely used to estimate unmeasurable variables. The available mathematical algorithms measure data to reconstruct unmeasurable variables or parameters of a system or a process. These kinds of systems are usually known as virtual sensors or soft sensors. Some applications of virtual sensors are: the monitoring of unknown variables or parameters of a mechanical system or a process [1–4]; the design of fault detection systems [5,6]; for fault detection isolation systems [7,8]; or for fault-tolerant control systems based on analytical redundancy [9]. In [10], a soft sensor is proposed using neural models in order to improve the product quality monitoring and control in a refinery. The advantages of the virtual sensors lie in that their implementation is easy, and in comparison with a hardware sensor, the cost is lower. In [11], the design and implementation of an observer-based soft sensor (virtual sensor) for a heat exchanger is presented.

The authors in [12] consider that the user interface is particularly important for educational software. There are some educational works concerning the heat exchanger [13,14] and thermodynamic systems [15,16], where the software is a teaching tool. The authors in [17] believe that learning can be enhanced by integrating the theoretical abstraction of textbooks with the tactile nature of the lab and plant.

Nowadays, the technological resources at universities and research departments allow one to develop sophisticated software with the aim of simulating the real conditions of the process. Most of the works in educational process are geared towards simulation [17–19]. However, developing practice or research in a pilot plant is more significant for the students, since they have the responsibility for the equipment and the process. Therefore, in this work, a monitoring and control interface for a heat exchanger pilot plant is presented.

## 2. The Heat Exchanger and Its Instrumentation

**Figure 1.**
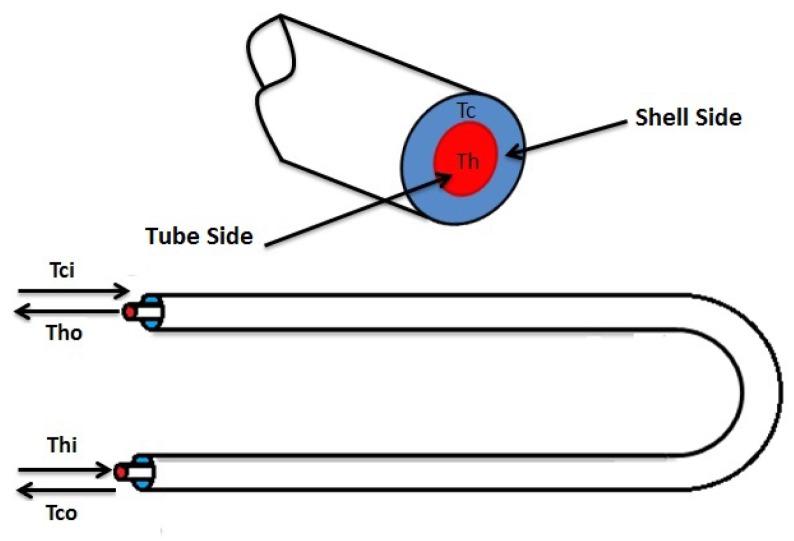
Countercurrent double-pipe heat exchanger.

A double pipe heat exchanger is a device where two fluids exchange heat. The double pipe heat exchanger is formed by two circular concentric pipes. The heat exchanger can be operated in parallel (the fluids flow in the same direction through the tube side and the shell side) or in counter-current flow (the fluids flow in opposite directions). In this work, the counter-current flow was used, as is shown in Figure 1.

The main variables involved in this process are:

- The inlet temperature of the fluid in the hot stream, *T_hi_*.
- The inlet temperature of the fluid in the cold stream, *T_ci_*.
- The outlet temperature of the fluid in the hot stream, *T_ho_*.
- The outlet temperature of the fluid in the cold stream, *T_co_*.
- The hot water flow, *W_vh_*.
- The cold water flow, *W_vc_*.

**Figure 2.**
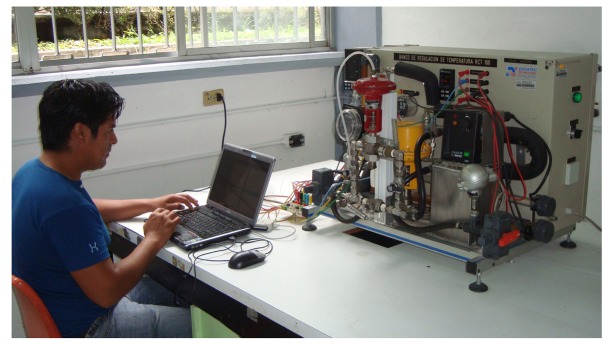
Heat exchanger pilot plant.

The double-pipe heat exchanger pilot plant produced by Didatec Technologies is located at the Process Control Laboratory of CENIDET (see Figure 2). The plant operates as a water-cooling process. In this heat exchanger, there is no change in the phase of any interacting fluid.

The pilot plant is provided with the following instrumentation:

- *T_ci_* and *T_ho_* are measured by two RTDPt-100 with four wires.
- *T_co_* and *T_hi_* are measured by two RTD Pt-100 with three wires.
- *W_vc_* and *W_vh_* are measured in two Platon variable section flow meters, and there is no digital signal from the flow meters.

The basic values of the Engelhard Pyro-Control Pt-100 temperature transmitter are given in Table 1. The instrumentation of the plant is sufficient to perform full variable monitoring, but not enough for automatic control tasks, since the flow rate cannot be recorded by the acquisition system. In the Didatec Technologies pilot-plant, the cold and hot water streams *W_vc_, W_vh_* are not measured by a digital transmitter (*W_vc_* is the manipulated variable, and *W_vh_* is taken as a constant). Furthermore, the system is not equipped with a human interface to allow the students to visualize, adequately, the data provided by the mentioned instruments and/or to control the plant actuators. Therefore, the students must be near the plant to visualize the measurements of the desired variables, which limits the user functions.

The goal of this work is to design a monitoring and control interface for the pilot plant in order to teach automatic control and heat transfer concepts. To develop the monitoring and control interface, an adequate acquisition system is required to transfer the plant's data to a computer, so it is necessary to integrate mathematic algorithms to estimate variables, such as temperature and flow rate.

### Heat Exchanger Modeling

In order to show the behavior of the main process variables in the heat transfer process and to develop a control system, a dynamic model was implemented. Furthermore, the algorithm model has implemented a thermodynamic model, which evaluates the density, heat capacity, convective heat transfer coefficients (*h_i_, h_o_*) and the global heat transfer coefficient; these values are displayed in a separate graphic.

The model was developed considering the follows conditions:

- A1. The flow is constant in the shell side.
- A2. The water physical properties are evaluated as functions of the temperature by empiric correlations.
- A3. The convective coefficient is dependent on the flow and temperature, and it varies with it.
- A4. There is no heat transfer between the outer pipe and the environment.
- A5. There is no energy accumulation in the pipe walls.
- A6. The system inputs are measurable.

The heat exchanger model is presented in Equation (1).

(1) $$\begin{array}{l} \frac{T_{co}}{dt}=\frac{W_{\upsilon c}}{V_{lc}}(T_{ci}(t)-T_{co}(t))+\left(\frac{UA_{o}}{C_{pc}\rho_{c}V_{lc}}\right)(T_{ho}(t)-T_{co}(t))\\ \frac{T_{ho}}{dt}=\frac{W_{\upsilon h}}{V_{lh}}(T_{hi}(t)-T_{ho}(t))+\left(\frac{UA_{i}}{C_{ph}\rho_{h}V_{lh}}\right)(T_{co}(t)-T_{ho}(t)) \end{array}$$

where *A_o_* and *A_i_* are the shell area and the tube side area, respectively. The thermodynamic model of the double-pipe heat exchanger was presented in [20].

## 3. The Data Acquisition System

The programming software selected to develop the monitoring interface is LabVIEW^©^, due to it being provided with algorithms to perform easily the remote monitoring tasks and to monitor the control systems, as well as having a user-friendly graphical interface, which is why a National Instruments (NI^©^) acquisition card was selected to communicate the heat-exchanger pilot plant information to the computer.

**Figure 3.**
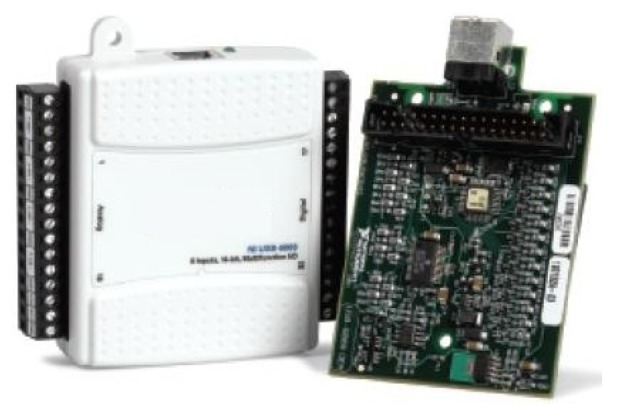
The selected National Instruments (NI) acquisition card.

The acquisition card used in this work is the NI USB-6008. It was selected because it is USB-compatible and has eight (12 bits) analog inputs, sufficient for acquiring all of the variables involved in the heat exchanger process; and it has two (12 bits) analog outputs used to send the data required to control the system actuators, as well as 12 digital I/O lines and a counter. The acquisition card is shown in Figure 3.

**Figure 4.**
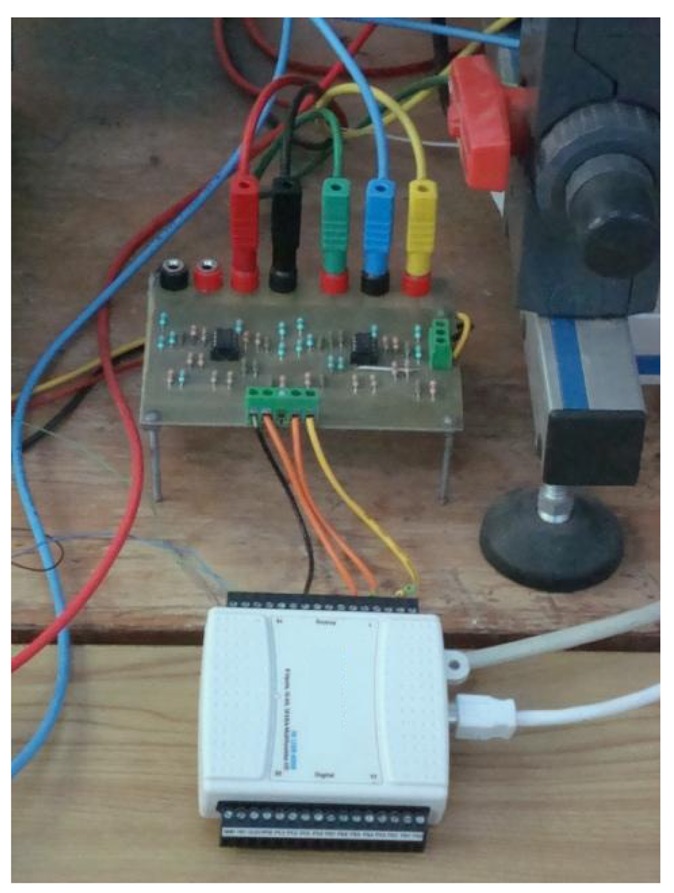
Connections and conditioning stage.

An advantages of this card is that it can be easily connected to the pilot plant, using a simple signal converter made by the students. The signal converter is used to convert the current signal (sensors signal) to the voltage signal (received by the DAQand then sending it to the computer), which is the input signal of the data acquisition. The connections of the signal converter are shown in Figure 4.

## 4. Design of the Monitoring and Interface

The designed interface is capable of performing three different tasks:

- Monitoring the inlet and outlet temperatures.
- Estimating the outlet temperatures, by using a heat exchanger model or by state observers to reconstruct a state variable from only one measure (*T_co_* or *T_ho_*) in order to implement a fault diagnosis scheme.
- Detecting and isolating sensor failures in the process with the aim of controlling the temperature, even if a failure occurs.

**Figure 5.**
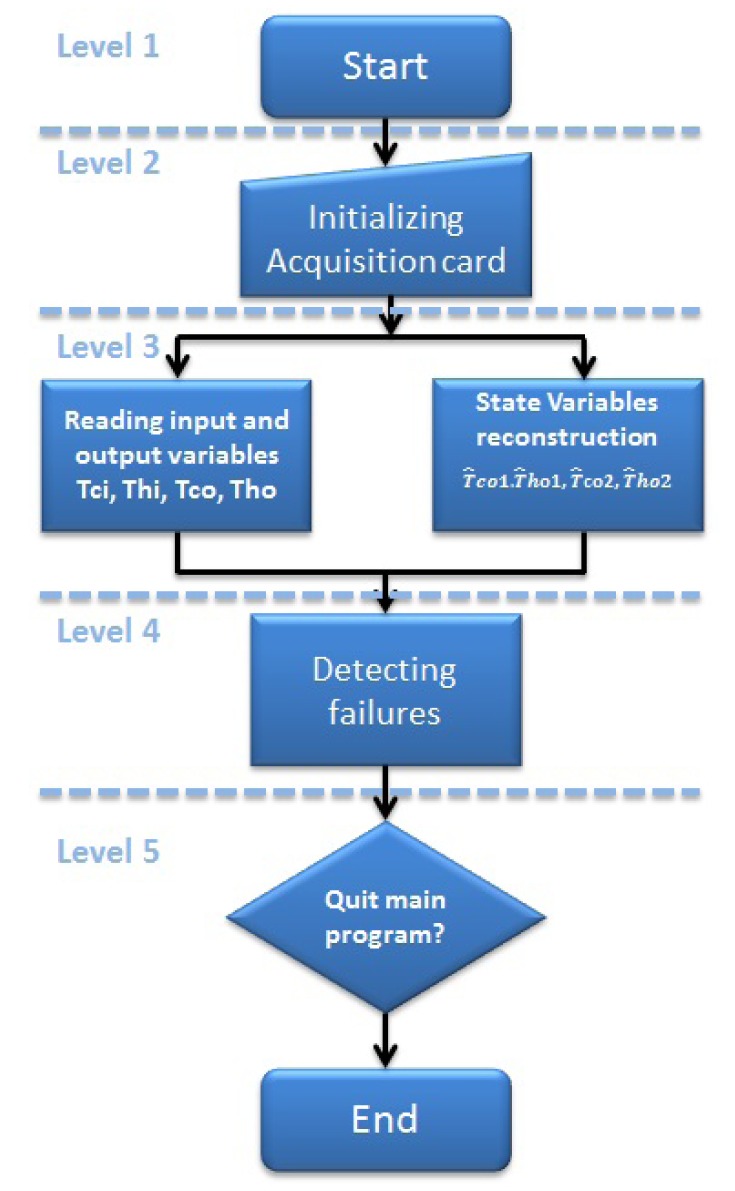
Hierarchic design.

These tasks are executed simultaneously, as can be seen in Figure 5.

The main program of the MCI system has a hierarchic design, which is shown in Figure 5. It provides features to select the subprogram to perform. Figure 5 shows the hierarchic design of the MCI.

Level 1: Initialization of the main program. LabVIEW^©^ has a running button to start the main program.
Level 2: Initialization of the data acquisition. In the MCI, there is an acquisition button.
Level 3: Signal reading and temperature estimation. The four temperature signals are read by the program, and these temperatures can be stored by a data store button, which is in the MCI. At the same level, there is the estimation of the temperatures, and at this level, a model and a bank of observers (virtual sensors) are programmed. The MCI has two buttons to request the model estimation and the virtual sensors estimation.
Level 4: The fault detection system, where the failure sensor could be replaced in case of failures.
Level 5: A quit button, which can be pushed to end the MCI.

An intuitive interface is required in order to perform the monitoring and control tasks in the heat exchanger pilot plant. Due this, the interface requires an adequate communication with the pilot plant. An acquisition card developed by NI^©^ is used to communicate data from/to the personal computer, which is why the LabVIEW^©^ software was selected.

As can be seen in Figure 5, a routine is necessary in order to initialize the settings required to acquire and send data to/from the pilot plant and from/to the personal computer. This routine is configured by the NI^©^ acquisition card, so it can be accessed by the computer using the indicated LabVIEW^©^ program. The main flow diagrams for the listed tasks are explained below.

**Figure 6.**
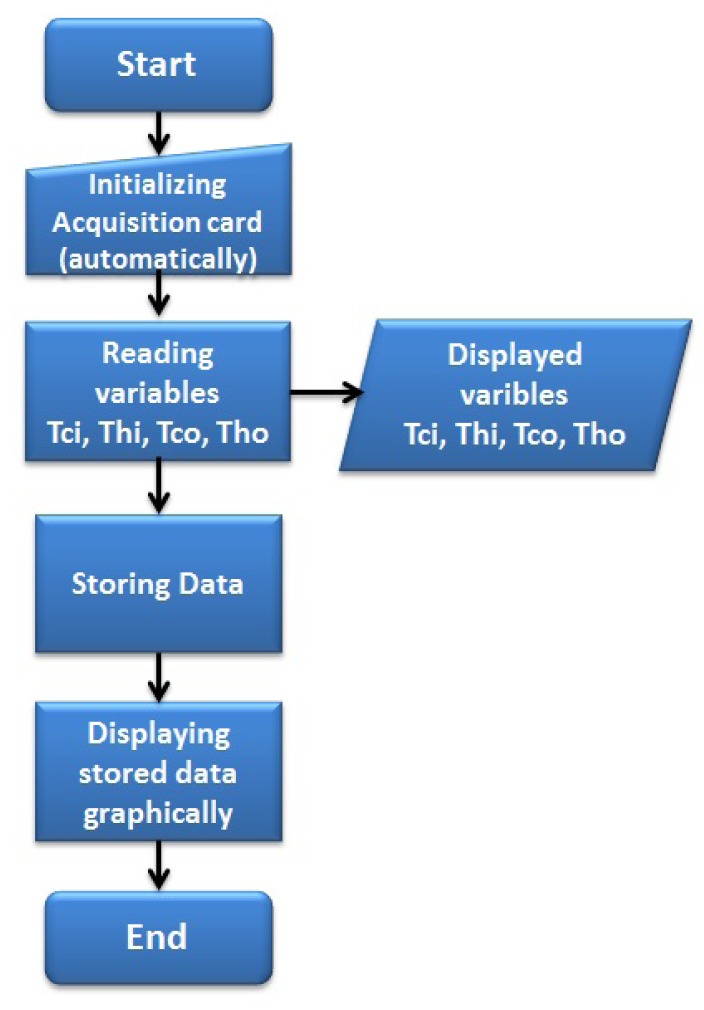
Variable monitoring.

**Figure 7.**
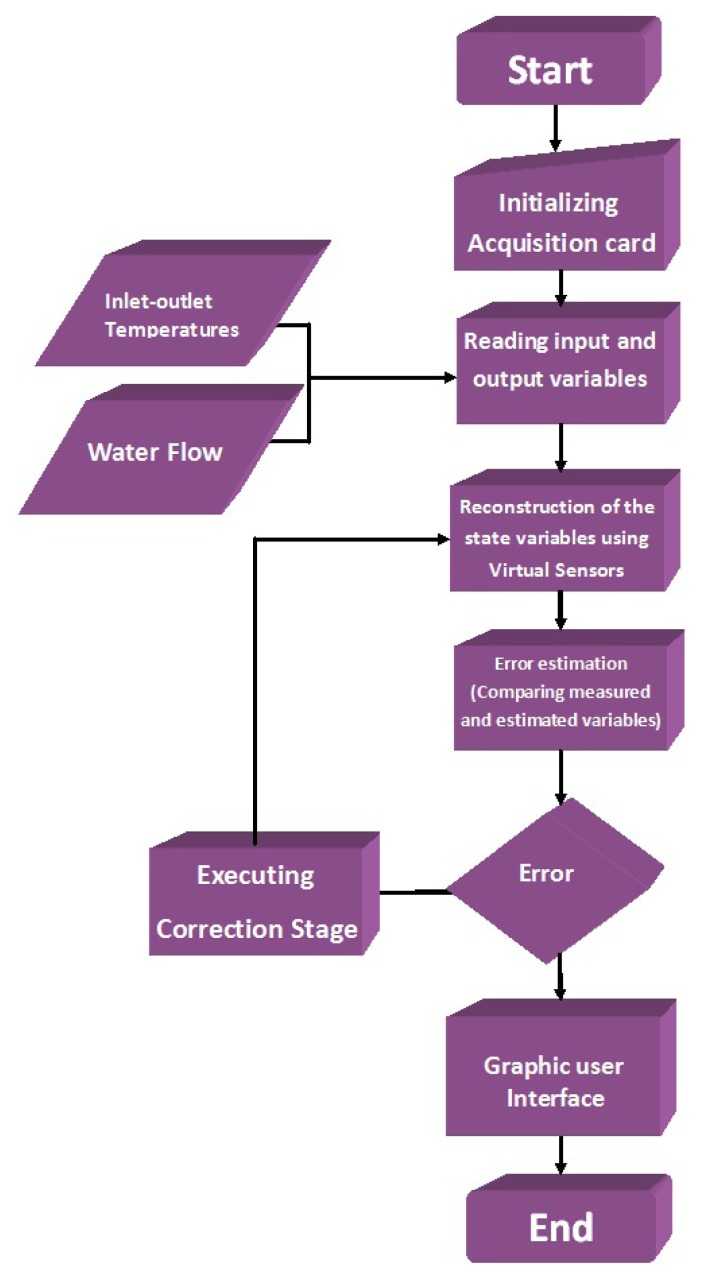
Estimating task.

**Figure 8.**
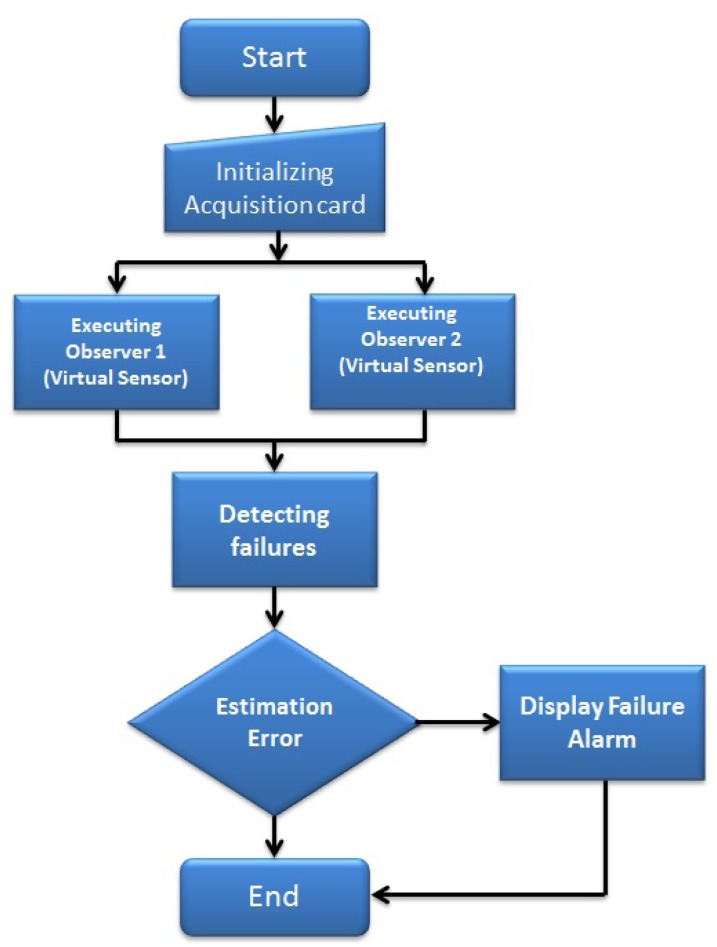
Failure detection function.

- Monitoring the temperatures plant: This algorithm reads a four-dimension data array, where the temperatures data are measured *T_ci_, T_hi_, T_co_, T_ho_* from the related sensors.
  The monitoring program (represented by the flow diagram; Figure 6) developed in LabVIEW^©^ allows the user to visualize the acquired data, through an on-line graphical interface. Different graphics are displayed for each temperature. The monitoring tasks are executed after the initializing routine automatically.
- Estimating the outlet temperatures (*T̂_co_* and *T̂_ho_*): This task executes an observer algorithm used to estimate both variables (*T̂_co_* and *T̂_ho_*) by using state observers to reconstruct a state variable from only one measure (*T_co_* or *T_ho_*) in order to implement a fault diagnosis scheme. The observer algorithm uses the input variables and the available output measure. It performs the required control algorithm, which, based on a model of the pilot plant, estimates the value of the two output variables (*T̂_co_* and *T̂_ho_*). The error is obtained by the comparison between the measured temperatures *T_co_* and *T_ho_* and the estimated temperatures *T̂_co_* and *T̂_ho_*. The flow diagram is shown in Figure 7.
  In the MCI, the temperature estimation (represented by the flow diagram shown in Figure 7) is executed by a button, which starts the estimation algorithms. The observer gain Equation (5) can be indicated in the interface or can be selected as a constant inside the program.
- Detecting sensor failures: An observer-based algorithm is used to detect and isolate failures in the sensors. Two observers are required, one for each physical sensor. Observer 1 uses *T_ci_, T_hi_, T_co_* as inputs, and Observer 2 uses *T_ci_, T_hi_, T_ho_* as inputs. The observers estimate the temperatures in the cold and hot stream (*T̂_co_, T̂_ho_*). A comparison is done between each temperature estimation and its corresponding physical sensor measure in order to evaluate the error (so, there are four error evaluations Equations (6)–(9)). The error is compared with an error tolerance, which is set by the user. If the error is bigger than the error tolerance, then an alarm is displayed in the MCI, indicating that a failure exists.
  Figure 8 shows the flow diagram that represents the fault detection realized by the virtual sensor or observer, and there is no interaction between the user and the interface when this task is running. In the case of a sensor failure, the MCI will display a green indicator.

## 5. Supervision and Control

### 5.1. Cold Water Flow Rate Solution

**Figure 9.**
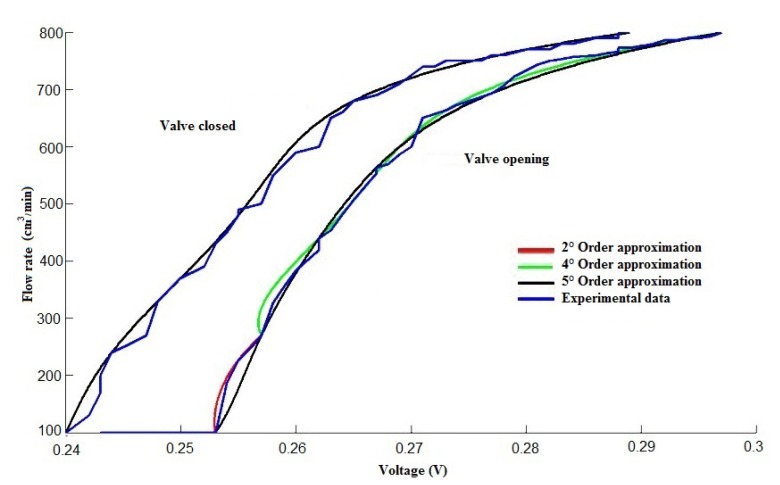
Valve characterization.

To monitor the heat exchanger control temperature using the cold water flow as the control input, it was necessary to characterize the control valve. In Figure 9, the valve characterization is shown, which presents hysteresis. Therefore, to send the control signal to the valve, it was convenient to use a Lagrange approximation (Equation (2)). The algorithm calculated the voltage necessary to control the valve according to the requested cold water flow (control input signal). It can be in open loop or in closed loop.

(2) $$\begin{array}{c} f(x_{k})=P_{n}(x_{k})k=0,1,2,\ldots ,n\\ P_{n}(x)=f(x_{0})L_{n,0}(x)+f(x_{1})L_{n,1}(x)+\cdots +f(x_{n})L_{n,n}(x)\\ L_{n,k}(x)=\frac{\left(X-X_{0})(X-X_{1})\cdots (X-X_{K+1})\cdots (X-X_{n}\right)}{\left(X_{K}-X_{0})(X_{K}-X_{1})\cdots (X_{K}-X_{K+1})\cdots (X-X_{n}\right)} \end{array}$$

where *x_k_* is the independent variable, *f*(*x_k_*) is the approximation of the independent variable, *L_n,k_*(*x*) is a polynomial base and *P_n_*(*x*) is the polynomial Lagrange interpolation.

### 5.2. Fault Detection and Isolation-Based Model

To accomplish the sensor fault detection and isolation (FDI), analytical redundancy was employed. To develop this system, a bank of nonlinear high gain observers was implemented. The bank implementation was done, because the fault detection system was designed to detect two possible failures, in the cold temperature sensor (*T_co_*) and in the hot temperature sensor (*T_ho_*), so it is necessary to have two virtual sensors (or a bank of virtual sensors) in order to replace any of the two physical sensors. In the next section, a general explanation of the FDI system is given.

#### Fault Detection and Isolation Nonlinear Approach

To implement the FDI system, a bank of observers was implemented. The design of the FDI based on a bank of nonlinear observers was studied in [20]. Therefore, in the present work, the equations are presented in a general form.

### 5.3. Control-Affine Nonlinear System

A control-affine nonlinear system can be formulated as Equation (3).

(3) $$\{\begin{array}{l} ẋ(t)=f(x(t))+\overset{m}{\underset{i=1}{\text{∑}}}g_{i}(x(t))u_{i}(t)\\ y(t)=h(x(t)) \end{array}$$

where *x*(*t*) ∈ ℝ*^n^, u_i_*(*t*) ∈ ℝ, *i* = 1,…,*m*, where *m* is the number of inputs, *y*(*t*) ∈ ℝ, *f*(*x*(*t*)) ∈ ℝ*^n^* and *g_i_*(*x*(*t*)) ∈ ℝ*^n^*, the last two are smooth vectors fields [20]. In these terms, if the model given in Equation (1) can be formulated as Equation (3), then it could be possible to design a nonlinear observer Equation (4).

### 5.4. Nonlinear Observer

The high gain nonlinear observer is defined by Equation (4).

(4) $$\{\begin{array}{l} \dot{\hat{x}}(t)=f(\hat{x}(t))+\overset{m}{\underset{i=1}{\text{∑}}}g_{i}(\hat{x}(t))u_{i}(t)-{\left[\frac{\partial \Phi (\hat{x}(t))}{\partial \hat{x}}\right]}^{-1}S_{\theta }^{-1}C^{T}[ŷ(t)-y(t)]\\ ŷ(t)=C\hat{x}(t) \end{array}$$

where *θ* > 0 is the tuning parameter of the observer and $\frac{\partial \Phi (x(t))}{\partial x}$ is the *n* x *n* Jacobian matrix of Φ(*x*(*t*)) and Φ(*x̂*(*t*)) = Φ(*x*(*t*))|*_x_*_(_*_t_*_)=_*_x̂_*_(_*_t_*_)_.

Considering a second order system, the matrix *S_θ_* is:

(5) $$S_{\theta }=\left[\begin{array}{cc} \frac{1}{\theta } & -\frac{1}{\theta^{2}}\\ -\frac{1}{\theta^{2}} & \frac{2}{\theta^{3}} \end{array}\right]$$

For the design of the FDI system, a bank of two observers is required.

### 5.5. Error Generation

The error evaluation between the physical sensors and the virtual sensors is obtained by Equations (6)–(9). An error evaluation is the difference between the measured temperature and the estimated temperature.

 Error evaluation from Observer 1:

(6) $$r_{11}=|T_{co}-{\hat{T}}_{co_{1}}|$$

(7) $$r_{12}=|T_{ho}-{\hat{T}}_{ho_{1}}|$$

Error evaluation from Observer 2:

(8) $$r_{21}=|T_{co}-{\hat{T}}_{co_{2}}|$$

(9) $$r_{22}=|T_{ho}-{\hat{T}}_{ho_{2}}|$$

where *T_ho_* is the hot water outlet temperature in the pipe and *T_co_* is the cooling water outlet temperature in the shell side. Therefore, the *T̂_co_* and *T̂_h__o_* are estimated values, and the subscript number (1 or 2) refers to the observer number.

According to the authors in [20], the switching between the measure and by Observer 1 or 2 depends on the failure (output sensor of the hot water or output sensor of the cooling water, respectively). Thus, the following conditions are given in order to perform the switching.

(10) $$T_{ho}=\{\begin{array}{ll} T_{ho} & \text{if}\;r_{12}<\zeta \Rightarrow \text{Normal operation}\\ {\hat{T}}_{ho} & \text{if}\;r_{12}\geq \zeta \Rightarrow \text{A failure exists} \end{array}$$

(11) $$T_{co}=\{\begin{array}{ll} T_{co} & \text{if}\;r_{21}<\zeta \Rightarrow \text{Normal operation}\\ {\hat{T}}_{co} & \text{if}\;r_{21}\geq \zeta \Rightarrow \text{A failure exists} \end{array}$$

*ζ* is error tolerance, which is a constant value and represents the maximum value of the error between the estimated variable by the virtual sensor and the real sensor signal value. Once this value is reached by any residual (*r*_12_, *r*_21_), the real sensor signal is switched, and it is replaced by the virtual sensor. This error tolerance value is set by the user.

### 5.6. Selection of a Control Law

For academic purposes, the MCI has implemented different control laws, which are Proportional (P) (Equation (12)), Proportional-Integral (PI) (Equation (13)), Proportional-Integral-Derivative (PID) (Equation (14)), MPC (Equation (18)) and a nonlinear control law-based model (Equation (23)). With the implementation of these control laws, the students can practice different tuning methods. Furthermore, the MCI allows the interaction between the FDI system and the control law to ensure a fault-tolerant system.

(12) $$P=K_{p}e(t)$$

(13) $$PI=K_{p}+K_{i}\int_{0}^{t}e(t)dt$$

(14) $$PID=K_{p}+K_{i}\int_{0}^{t}e(t)dt+Kd\frac{de}{dt}$$

To develop the prediction algorithm for the MPC, the state-space model given in Equation (15) was considered. The development of MPC is presented in detail in [21].

(15) $$\begin{array}{cc} {\text{x}}_{k+1} & ={\text{Ax}}_{k}+{\text{Bu}}_{k}\\ y_{k+1} & ={\text{Cx}}_{k}+{\text{Du}}_{k} \end{array}$$

where *x* is the state vector, *y* is the output vector and *u* is the input vector.

(16) $$\begin{array}{cc} \frac{d\bar{x}}{dt} & =\text{A}\bar{x}+\text{B}ū\\ \bar{y} & =\text{C}\bar{x}+\text{D}ū \end{array}$$

In Equation (16), *x̄* denotes that the systems is linearized at one point. For linear systems of the form given in Equation (16), the development of the prediction model is simple:

(17) $$\begin{array}{ll} {\text{x}}_{k+n} & ={\text{A}}^{n}{\text{x}}_{k}+{\text{A}}^{n-1}{\text{Bu}}_{k}+{\text{A}}^{n-2}{\text{Bu}}_{k+1}+\cdots +{\text{Bu}}_{k+n-1}\\ y_{k+n} & =\text{C}[{\text{A}}^{n}{\text{x}}_{k}+{\text{A}}^{n-1}{\text{Bu}}_{k}+{\text{A}}^{n-2}{\text{Bu}}_{k+1}+\cdots +{\text{Bu}}_{k+n-1}] \end{array}$$

To develop the control algorithm in the absence of input or output constraints, a generalized predictive control law is reduced to a linear feedback control strategy. State-space systems based on the predictive control law take the form:

(18) $$\text{u}-{\text{u}}_{ss}=-k(\text{x}-{\text{x}}_{ss})$$

A model-based nonlinear control law proposed in [22] was also implemented. The nonlinear control law Equation (23) is based on the nonlinear model of the heat exchanger Equation (1).

(19) $$ё={ÿ}_{m}-c\left(\frac{\partial f}{\partial x}\right)\text{ẋ}$$

(20) $$\frac{\partial f}{\partial x}=\left[\begin{array}{cc} \frac{1}{V_{lc}}\left({Ẇ}_{\upsilon c}(T_{ci}-T_{co})-W_{\upsilon c}\right)+\frac{UA}{\rho_{c}Cp_{c}V_{lc}}\frac{\partial \Delta Tml}{\partial x_{1}} & \frac{UA}{\rho_{c}Cp_{c}V_{lc}}\frac{\partial \Delta Tml}{\partial x_{2}}\\ -\frac{UA}{\rho_{h}Cp_{h}V_{lh}}\frac{\partial \Delta Tml}{\partial x_{1}} & \frac{W_{\upsilon h}}{V_{l}h}-\frac{UA}{\rho_{c}Cp_{c}V_{lc}}\frac{\partial \Delta Tml}{\partial x_{2}} \end{array}\right]$$

(21) $$ė=\lambda_{m}y_{m}-\lambda_{m}y_{s}$$

(22) $$ё=-k\left(e+\frac{1}{\tau_{i}}\int_{0}^{t}e\;dt+\tau_{d}\;ė\right)$$

(23) $$W_{\upsilon h}=\frac{\frac{1}{V_{lc}}\left({Ẇ}_{\upsilon c}(T_{ci}-T_{co})-W_{\upsilon c}{Ṫ}_{co}\right)+\frac{UA}{\rho_{c}Cp_{c}V_{lc}}\left[\frac{\partial \Delta Tml}{\partial x_{1}}\frac{UA}{\rho_{h}Cp_{h}V_{lh}}\Delta Tml\right]+ё}{\frac{UA}{\rho_{c}Cp_{c}V_{lc}}\frac{\partial \Delta Tml}{\partial x_{2}}\left(T_{hi}-T_{ho}\right)}$$

The graphical user interface displays the parameters of the selected control law.

## 6. The Monitoring and Control Interface Implementation

**Figure 10.**
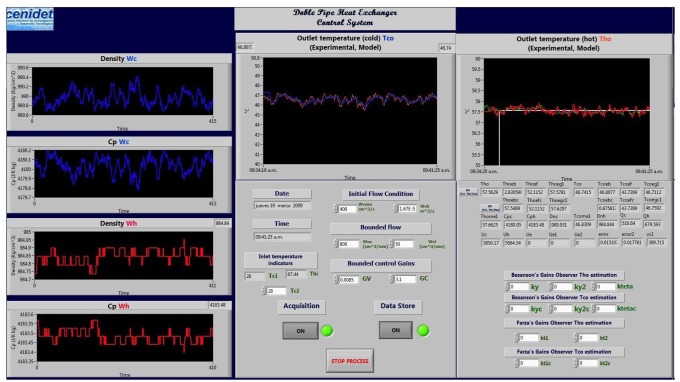
Main function graphical interface.

The MCI was programed in order to realize sensors fault detection and isolation and fault tolerance using virtual sensors. The virtual sensors were designed from model-based high-gain observers. To develop the control task, different kinds of control laws were included in the monitoring and control interface. These control laws are PID, MPC and a nonlinear model-based control law. The MCI helps to maintain the heat exchanger under operation, even if a temperature outlet sensor fault occurs; in the case of outlet temperature sensor failure, the MCI displays an alarm. The monitoring and control interface is used as a practical tool to support electronic engineering students with heat transfer and control concepts to be applied in a double-pipe heat exchanger pilot plant. The method aims to teach the students through the observation and manipulation of the main variables of the process and by the interaction with the monitoring and control interface (MCI) developed in LabVIEW^©^. In this section, the monitoring and control interface is presented (Figure 10), which was developed in LabVIEW^©^, and some control algorithms of the MCI are shown.

The MCI allows the students to develop and implement approximation algorithms. The MCI is a tool that facilitates the interaction between the students and the process (heat exchanger). This tool allows students to understand the control algorithms that were programmed in the MCI. Furthermore, students can interact with the MCI in different ways, such as monitoring the states of the process, tuning the PID control law and developing control algorithms using the MATLAB programming language. At CENIDET, there are courses on MATLAB to train the students in programming skills. A common practice is to ask the students to develop a program for the heat exchanger's model using the MATLAB programming language, in order to realize a comparison with the real temperature signals acquired.

In Figure 10, the density and heat capacity of the fluid in both streams of the heat exchanger are shown (left-most section; tube side and shell side). In the upper center section of Figure 10, the cooling water temperature is shown (the red line shows the measured temperatures, and the blue line shows the estimated temperatures). In the right side of Figure 10, the hot water temperature is shown (the green line shows the measured temperatures, and the red line shows the estimated temperatures). The interface is intuitive for the users. Each section is adequately signposted, and it is continuously enhanced by the students according to their needs.

**Figure 11.**
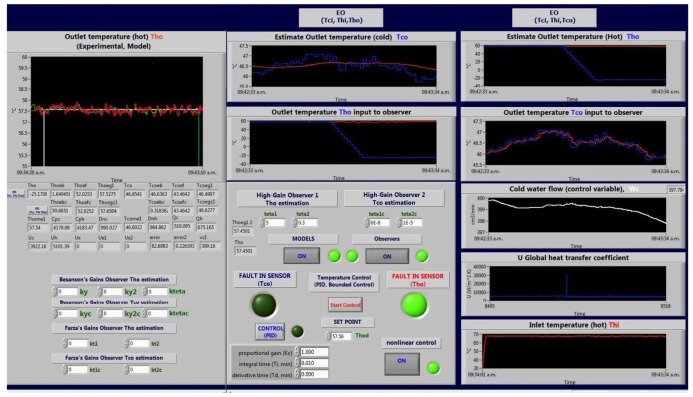
Fault-tolerant system.

In Figure 11 (left side), the hot water temperature is shown (the green line shows measured temperature, and the red line shows the estimated temperatures). In the middle of the figure, the fault detection and control section are shown. As can be seen, the green light is lit, because of a fault in a hot water temperature sensor (total failure in the sensor). However, the system continues its operation, because the measured temperature is replaced by the estimated temperature (by *observer*_1_), which does not depends on the *T_ho_* measure for temperature. Other graphics are shown in this figure, such as the observer estimation (on the top), the control variable (cooling water flow) and the global heat transfer coefficient.

**Figure 12.**
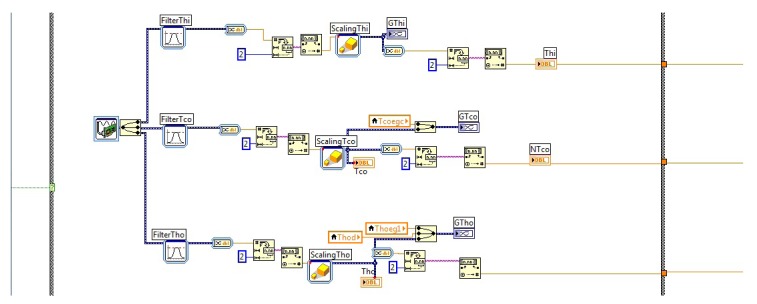
Acquisition algorithm using LabVIEW^©^.

Figure 12 shows the program of the monitoring and control interface. In Figure 12, the data acquisition algorithm using LabVIEW^©^ is presented. For this work, three temperatures were measured and acquired for the instrument *T_hi_, T_co_* and *T_ho_*.

**Figure 13.**
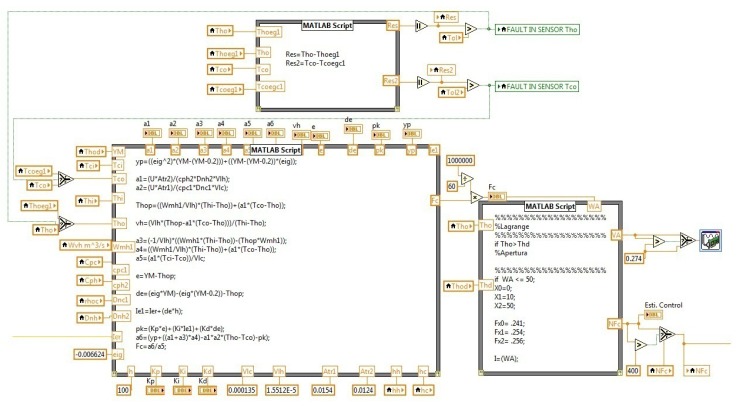
Fault detection algorithm.

At the top of the Figure 13, the error evaluation and the alarms section are shown. In the bottom of the figure (Figure 13), the nonlinear control law is shown, which was implemented for the closed loop; also, it has the Lagrange approximation to perform the closed loop control.

**Figure 14.**
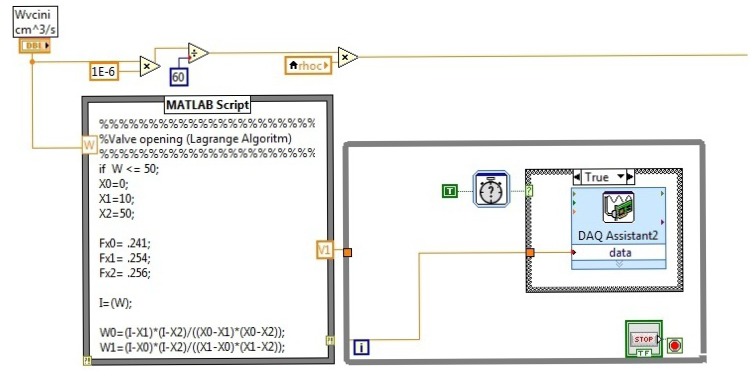
Open loop algorithm.

In Figure 14, the open loop control algorithm is shown for manual operation. The user can change the cold water flow manually through a slide manipulation, which is on the front panel of the MCI.

**Figure 15.**
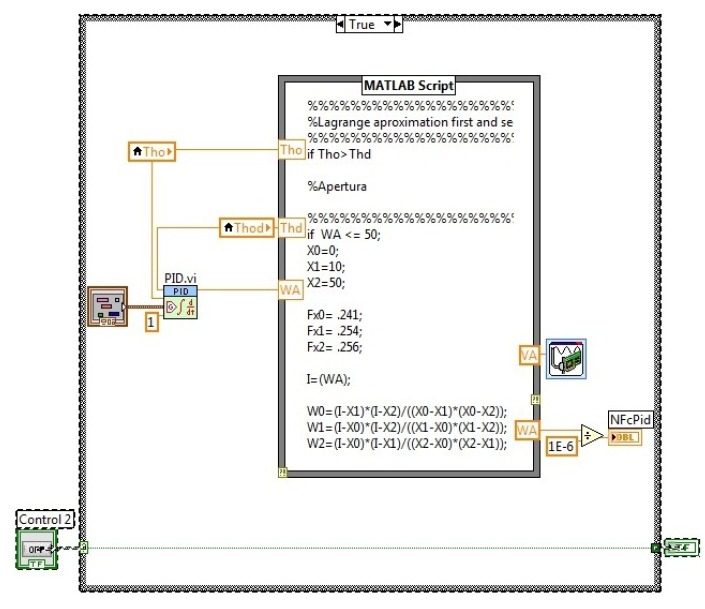
Closed loop algorithm.

The figure shows the Lagrange approximation, which is used to calculate the required voltage for the control flow. Figure 15 shows the PID controller; also, it has the Lagrange approximation to perform the closed loop control.

## 7. Results

With the monitoring control interface (MCI), the instructors for the control process, nonlinear control and classic control subjects have the support to develop their laboratory practices. Furthermore, most of the students can easily interact with the heat exchanger, even if they have little experience in heat exchange devices. The results obtained with the design of the MCI are: 100% of the EES that belong to the automatic control area have had the opportunity to practice at least once with the heat exchange devices during their stay at CENIDET. One student is developing his research on fault-tolerant control with an application in a heat exchanger. Another student is developing his research project on the design of state observers applied in a heat exchange process.

**Figure 16.**
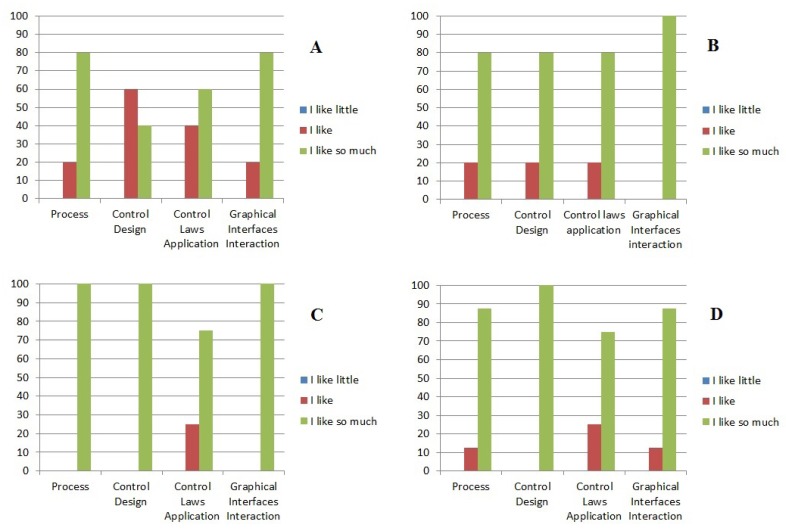
Statistics on the use of the monitoring and control interface (MCI).

Figure 16 shows the opinion of the students about the MCI. In Figure 16, Sections A and B are the contents for the students that have made use of the MCI. In Figure 16, Sections C and D are the contents for students which have no made use of the MCI. We formulate two questions for the students that have made use of the MCI. These questions have four different concepts included. Question 1: How much has the use of the monitoring and control interface allowed you to assimilate clearly the following criteria? Question 2: How much has the use of the monitoring and control interface awakened your interest with respect to the following criteria? The criteria for both cases were: Process, control design, control law application and graphical interface interaction. In Sections C and D of Figure 16, the questions applied were as follows. Question 1: How much has the use of the interface enabled you to assimilate the following criteria? Question 2: How much has the use of monitoring and control interface sparked your interest in the following criteria?

Since the MCI has been in use, the interest of the students in heat exchanger control has increased, because, during the course period, the EES have acquired experience and practice in the operation of these devices. Furthermore, they have acquired knowledge in the control area and for how they can develop their control algorithms to design soft sensors or other control techniques.

CENIDET is the National Center for Research and Technological Development, which provides educational training support to technological universities. Students from 15 or 20 (approximately) technological universities of Mexico carry out professional practice in CENIDET, and in most of the cases, these students select CENIDET to develop their master studies. Some of the students that have had professional practice using the MCI and who are now working on their Master's thesis project are: Diego Alessis Carbot Rojas, “Actuator fault-tolerant control: Application in a heat transfer process”; Juan Pablo Castillo González, “Control design for a biodiesel reactor”; and Moisés Bulmaro Ramos Martínez, “Control design for an azeotropic process”. Other success cases in the implementation of the MCI are students that decide to continue their Doctoral studies, students such as: Betty Yolanda López Zapa “Fault diagnosis applied in a biodiesel reactor”; Jarniel García Morales “Multiple-fuel control design for an internal combustion engine”; and Omar Hernández González “Observation and control of nonlinear process systems for energy recovery from waste heat”. In addition, students have published articles in the control area using the heat exchanger device and the knowledge that the MCI provides: the most recent work done by a student is under review, however, in the *Revista Mexicana de Ingeniería Química*, this work is entitled Multiple Sensor Fault Diagnosis in a Heat Exchanger Using Sliding-Mode Observers Based on Super-Twisting Algorithm. Another example of the contribution made by the students is given in the work presented in [23], where the student, Betty Yolanda López-Zapata, was a collaborator. Furthermore, the MCI has provided support to develop some published works regarding heat exchanger devices and their control [24,25].

## 8. Conclusions

The monitoring control interface (MCI) is an excellent alternative to implement in a real heat exchange process in order to make the system fault tolerant. The MCI is capable of detecting and isolating failures using virtual sensors. These virtual sensors are accurate in outlet temperatures estimation. Furthermore, the MCI is an excellent option to strengthen the knowledge taught in the classroom. Subjects, such as classic control, nonlinear control and fault detection and isolation (via two different approaches, linear and nonlinear observers), are supported by this interactive educational workstation. The participation and the interest of the students in laboratory practices employing the heat exchanger pilot plant increased by more than 20% since the MCI was implemented, according to a survey. In a similar way, the lecturers were very interested in developing other algorithms to enrich the control area. For future work, subjects, such as fuzzy control, neural networks and system identification, could be added to the interactive educational workstation.

## Figures and Tables

**Table 1.** RTDPt-100 values.

***RTD***	**≤±0.2 ^°^C**	**≤±0.01 °C/°C**
***Lin.R***	**≤±0.1 Ω**	**≤±0.01 mΩ/°C**
***V olt***	**≤±10 μV**	**≤±1 mμ/°C**
Temperature range RTD 3W		−40 °C to 85 °C
Temperature range RTD 4W		0 °C to 100 °C
Effect of supply voltage variation		<0.005% of span/VDC
Vibration		IEC 60068-2-6 Test FC
Max. wire size		1 x 1.5 mm^2^ stranded wire
Dimensions		44 x 20.2 mm
Weight		50 g
Max offset		50% of selec.max value
Cable resistance per wire (max)		5 Ω
Output sensor current		0.2…0.4 mA
Effect of sensor cable resistance (3-/4-wire)		<0.002 Ω/Ω

## Appendix A

*Nomenclature*	
*T*	Temperature, (^°^C)
*U*	Heat transfer coefficient, (W/m^2^K)
*A*	Heat transfer area, (m^2^)
*C_p_*	Specific heat, (J/kg K)
*V_l_*	Volume, (m^3^)
*W_v_*	Volumetric flow, (cm^3^/min)

*Greek letters*	
*ρ*	Density, (kg/m^3^)
*ζ*	Error tolerance
*θ*	Observer gain
Δ*T*	Temperature increment
*λ*	Eigenvalue
*ζ*	Error tolerance

*Subscripts*	
*c*	Cold
*h*	Hot
*i*	Input
*o*	Output
*ss*	Steady state
